# Modeling the elevated risk of yellow fever among travelers visiting Brazil, 2018

**DOI:** 10.1186/s12976-018-0081-1

**Published:** 2018-07-02

**Authors:** Yohei Sakamoto, Takayuki Yamaguchi, Nao Yamamoto, Hiroshi Nishiura

**Affiliations:** 1 Graduate School of Medicine, Hokkaido University, Kita 15 Jo Nishi 7 Chome, Kita-ku, Sapporo-shi, Hokkaido 060-8638 Japan; 2 CREST, Japan Science and Technology Agency, Honcho 4-1-8, Kawaguchi, Saitama, 332-0012 Japan

**Keywords:** *Flavivirus*, Traveler, Importation, Risk assessment, Epidemic, Vaccination

## Abstract

**Background:**

Unlike the epidemic of yellow fever from 2016 to 17 in Brazil mostly restricted to the States of Minas Gerais and Espirito Santo, the epidemic from 2017 to 18 mainly involved São Paulo and Rio de Janeiro and resulted in multiple international disseminations. To understand mechanisms behind this observation, the present study analyzed the distribution of imported cases from Brazil, 2018.

**Methods:**

A statistical model was employed to capture the risk of importing yellow fever by returning international travelers from Brazil. We estimated the relative risk of importation among travelers by the extent of wealth measured by GDP per capita and the relative risk obtained by random assignment of travelers’ destination within Brazil by the relative population size.

**Results:**

Upper-half wealthier countries had 2.1 to 3.4 times greater risk of importation than remainders. Even among countries with lower half of GDP per capita, the risk of importation was 2.5 to 2.8 times greater than assuming that the risk of travelers’ infection within Brazil is determined by the regional population size.

**Conclusions:**

Travelers from wealthier countries were at elevated risk of yellow fever, allowing us to speculate that travelers’ local destination and behavior at high risk of infection are likely to act as a key determinant of the heterogeneous risk of importation. It is advised to inform travelers over the ongoing geographic foci of transmission, and if it appears unavoidable to visit tourist destination that has the history of producing imported cases, travelers must be strongly advised to receive vaccination in advance.

## Background

Yellow fever virus that belongs to *Flavivirus* causes yellow fever, transmitted via *Aedes* species from infected human to human [1]. In addition to human-mosquito-human transmission cycle, nonhuman primates are also infected with the virus, and such transmission cycle has been known to be responsible for allowing continued transmission in Brazil [2–4]. The exposure to this virus mostly results in asymptomatic infection, but a part of cases develop fever, headache, chills, muscle pain, nausea and vomiting following an incubation period of 3–6 days [1]. If exacerbated, the case fatality risk (CFR) given severe clinical disease is known to be 47% with the range from 40 to 80% [5], and there is no specific treatment. Thus, immunization is the mainstream of countermeasures [6], and residents of high-risk area and travelers visiting those areas are recommended to undertake vaccination.

The epidemic of yellow fever in Brazil from December 2016 to June 2017 involved 777 confirmed cases, all reported among Brazilian residents, and the CFR was estimated at 34% with 261 deaths [7]. While the end of epidemic was declared once in September 2017, sporadic cases continued and a surge of cases started from December 2017 [8]. On 16 January 2018, World Health Organization (WHO) recognized the ongoing epidemic and recommended vaccination among all residents of the entire State of Rio de Janeiro and São Paulo, and vaccination campaign by the government of Brazil took place with targeting States Bahia, Rio de Janeiro, São Paulo [8]. As of 8 June 2018, 1257 confirmed cases and 394 deaths have been reported [9].

While no imported case was reported during the epidemic from 2016 to 17, multiple importation events have been reported during the epidemic from 2017 to 18. There have been 12 imported cases, as of 8 June 2018, notified in 8 different countries since December 2017 [9, 10]. As a possible mechanism for observing multiple importation events, the epidemic location in 2017–18 has involved Rio de Janeiro and São Paulo more than the 2016–17 epidemic [11]. The present study aims to quantify the risk of infection among travelers visiting Brazil, 2018.

## Methods

### Epidemiological data

To estimate the risk of yellow fever among travelers, we analyze both the confirmed cases in Brazil from 2017 to 18 [9] and imported cases reported abroad. As of 8 May 2018, there were 3 imported cases from Chile [12], 3 from Argentina [12], and 1 case from the Netherlands [12], Switzerland [13], France [12], the United Kingdom [10], Romania [13] and Germany [14], respectively. Nationality of all imported cases was their own country, and all these cases were regarded as importation due to travel to Brazil. Except for cases in the Netherlands and France and one case in Argentina, imported cases shared a history of visit to Ilha Grande, municipality of Angra do Reis, State of Rio de Janeiro, Brazil [12].

### Mathematical model

To calculate the expected risk of importation, inbound travel volume *c*_i_ from each country *i* to Brazil was retrieved from the World Tourism Organization [15]. In addition, we used the relative value of the Gross Domestic Product (GDP) per capita, *g*_i_ of country *i* that was normalized by the maximum GDP in 2016 for the sake of imputation of vaccination coverage (see below) [16]. This imputation was partly validated by statistical analysis of an association between GDP per capita and the risk of importation by country. Confirming that the variance is not significantly different between two groups by F-test, we employed Student t-test to compare the GDP per capita between countries with and without imported cases. Moreover, vaccination coverage *v*_i_ of country *i* in 2015 was partly retrieved from a published study [17].

Following Dorigatti et al. [18], we model the expected number E(*c*_i_) of imported yellow fever cases *c*_i_ in country *i* as

1 $$ E\left({c}_i\right)={n}_i\left(1-{v}_i\right){q}_i\frac{pop_S}{pop_B}\frac{c_S}{pop_S}\frac{\mu_E+{\mu}_I}{w}\sum \limits_{s=1}^{12}{f}_s{p}_s $$

In this equation, *n*_i_ is the yearly inbound number of travelers visiting Brazil from country *i*, *v*_i_ is the vaccination coverage, *q*_i_ represents the relative risk of infection among travelers from country *i*, which we would like to estimate through this exercise. *pop*_s_ and *pop*_B_ represent the population sizes of the three major states of 2017–18 epidemic (i.e., Minas Gerais, Rio de Janeiro and São Paulo) and the entire Brazil, respectively (*pop*_s_ = 81,230,574 and *pop*_B_ = 202,768,562 persons). The reported number of confirmed yellow fever cases in affected states is *c*_S_. We do not use the undiagnosed factor of 10, which was adopted elsewhere [5, 18], because we estimate the expected number of confirmed imported cases in the abroad. *w* is the mean length of stay in Brazil (w = 17 days), and *f*_s_ and *p*_s_ are the normalized monthly frequency of cases and the volume of travelers of month *s*, respectively (where *f*_s_ from December 2017 to March 2018 accounted for 98.4% and *p*_s_ during the same period is 47.6% of the total). Although not specified in eq. (1), month *s* was integrated from December 2017 up until 16 March 2018 where censoring in March was incorporated by accounting for the number of days, i.e., 16/31. μ_E_ and μ_I_ represent the mean latent and infectious period, respectively, assumed as 4.6 and 4.5 days. It should be noted that eq. (1) is intact even when we account for unascertained/asymptomatic fraction of cases. Namely, supposing that the confirmation probability among all infected individuals is *α*, both sides of eq. (1) is divided by *α* to express everything as the total number of infected individuals, and then, the constant 1/*α* is cancelled out from both sides.

Unlike calculations that were conducted elsewhere [18, 19], we ignored the stochasticity of the lengths of latent and infectious periods for simplicity. Let us define

2 $$ {m}_i\left({q}_i\right):= \frac{E\left({c}_i;{q}_i\right)}{1-{v}_i}, $$

we employ the zero-inflated Poisson distribution to describe the observed frequency of imported cases in country *i*, i.e.,

3 $$ h\left(X=j;{q}_i\right)=\left\{\begin{array}{l}{v}_i+\left(1-{v}_i\right)\exp \left(-{m}_i\left({q}_i\right)\right)\kern2.5em \mathrm{if}\ j=0\\ {}\left(1-{v}_i\right)\frac{\exp \left(-{m}_i\left({q}_i\right)\right){m}_i{\left({q}_i\right)}^j}{j!}\kern1.25em \mathrm{if}\ j>0\end{array}\right. $$

The mean number of imported cases from the eq. (3) is E(*c*_i_) = (1-*v*_i_)*m*_i_(*q*_i_). With respect to the vaccination coverage *v*_i_ among travelers from country *i*, we model it as

4 $$ {v}_i=\left\{\begin{array}{l}{u}_i\kern4.75em \mathrm{if}\ \mathrm{vaccination}\ \mathrm{coverage}\ \mathrm{available}\\ {}\frac{k}{1+\exp \left(-{g}_i\right)}\kern0.5em \mathrm{if}\ \mathrm{vaccination}\ \mathrm{coverage}\ \mathrm{unavailable}\end{array}\right. $$

The logit transformation was adopted, because it does not require additional parameters. The estimated vaccine coverage in a part of the routinely immunized countries was available in the published study [17]. Countries with known vaccination coverage included Trinidad and Tobago, Panama, Argentina, Colombia, Suriname, Peru, Venezuela, Ecuador, Paraguay, Guyana, Bolivia, Angola, Nigeria, Ghana and Kenya. If the information of vaccination coverage was unavailable, we extrapolated the vaccination coverage in country *i*. Considering that only a small fraction of travelers is vaccinated in those countries, *k* was assumed to be 0.10, which is the so-called carrying capacity of the logistic distribution, and practically interpreted as theoretical possible maximum of the vaccination coverage. *g*_i_ is the relative GDP per capita of country *i* compared to the country with the highest GDP per capita as stated above. The second case in the eq. (4) indicates that we imposed an assumption that, if no routine immunization takes place, at most 10% of those travelers visiting Brazil received vaccination, and also another assumption that the coverage follows a logit transformation of the relative GDP per capita. Because the ceiling of coverage *k* is a strong assumption, we varied it from 0.01 (1%) to 0.90 (90%) as part of sensitivity analysis.

We estimate *q*_i_ by the list of country, i.e. by first and second half of GDP per capita, because the propensity to visit high-risk area of infection, which coincided with a resort area in the ongoing epidemic [10, 12], may vary with the extent of wealth of that particular country. *q*_i_ is modelled as

5 $$ {q}_i=\left\{\begin{array}{l} aq\kern1.75em \mathrm{if}\ \mathrm{GDP} per\  capita\ \mathrm{of}\ \mathrm{country}\ i\ \mathrm{is}\ \mathrm{greater}\ \mathrm{than}\ \mathrm{median}\\ {}q\kern2.5em \mathrm{if}\ \mathrm{GDP} per\  capita\ \mathrm{of}\ \mathrm{country}\ i\ \mathrm{is}\ \mathrm{smaller}\ \mathrm{than}\ \mathrm{median}\end{array}\right. $$

where *q* is the relative risk of infection among travelers visiting from countries with the second half of GDP per capita as compared with an assumption that the travelers’ destination is randomly determined according to the regional population size of Brazil. *a* is the relative risk of importing yellow fever among wealthier countries compared to the reminders with the risk *q*.

Given observed counts of imported cases, **c**, written as a vector representing the input from all countries at risk of infection, maximum likelihood estimates of *a* and *q* were found by minimizing the negative logarithm of the following likelihood

6 $$ L\left(a,q;\mathbf{c}\right)=\prod \limits_ih\left({c}_i\right) $$

The 95% confidence intervals (CI) were computed using the profile likelihood.

### Ethical considerations

The present study analyzed data that is publicly available. As such, the datasets used in our study were de-identified and fully anonymized in advance, and the analysis of publicly available data without identity information does not require ethical approval.

## Results

**Fig. 1:**
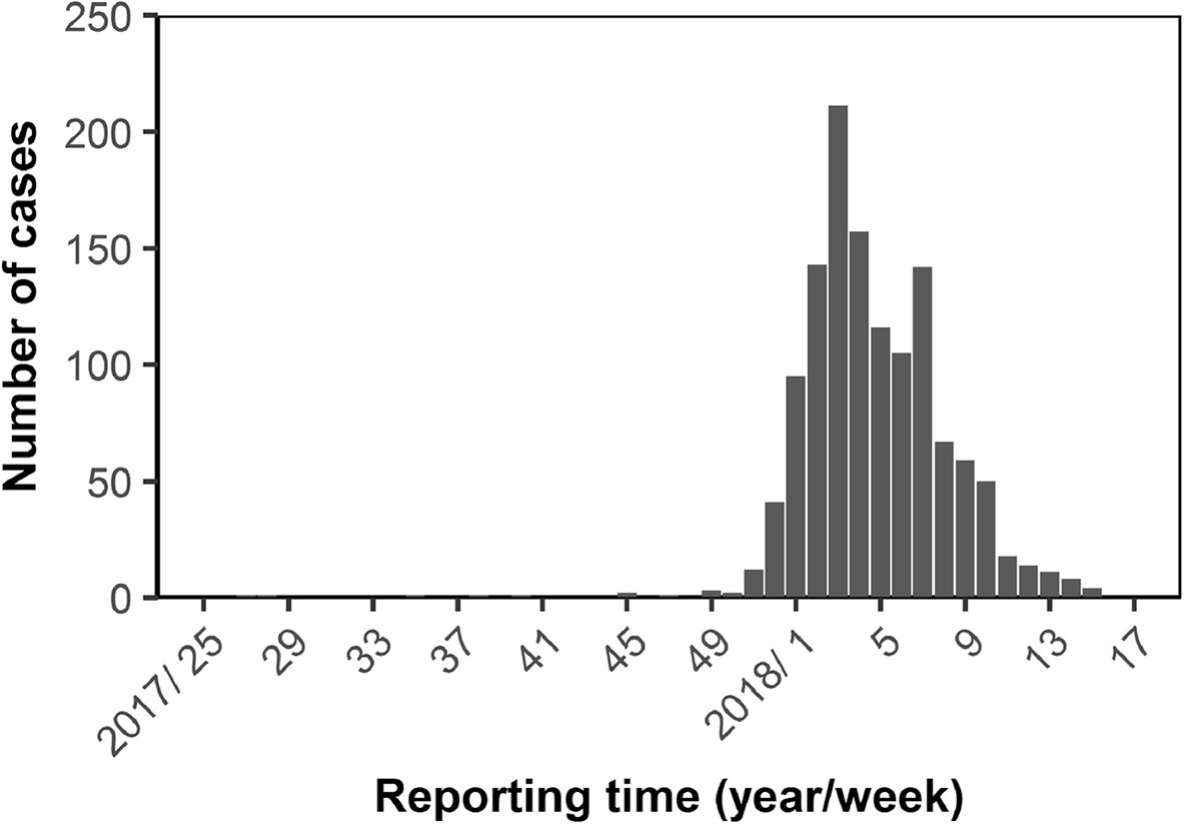
Weekly incidence of the yellow fever cases in Brazil from 2017 to 18. Weekly count of confirmed cases is reported as a function of the week of report [9]. The highest incidence was reported on Week 3 of 2018

**Fig. 2:**
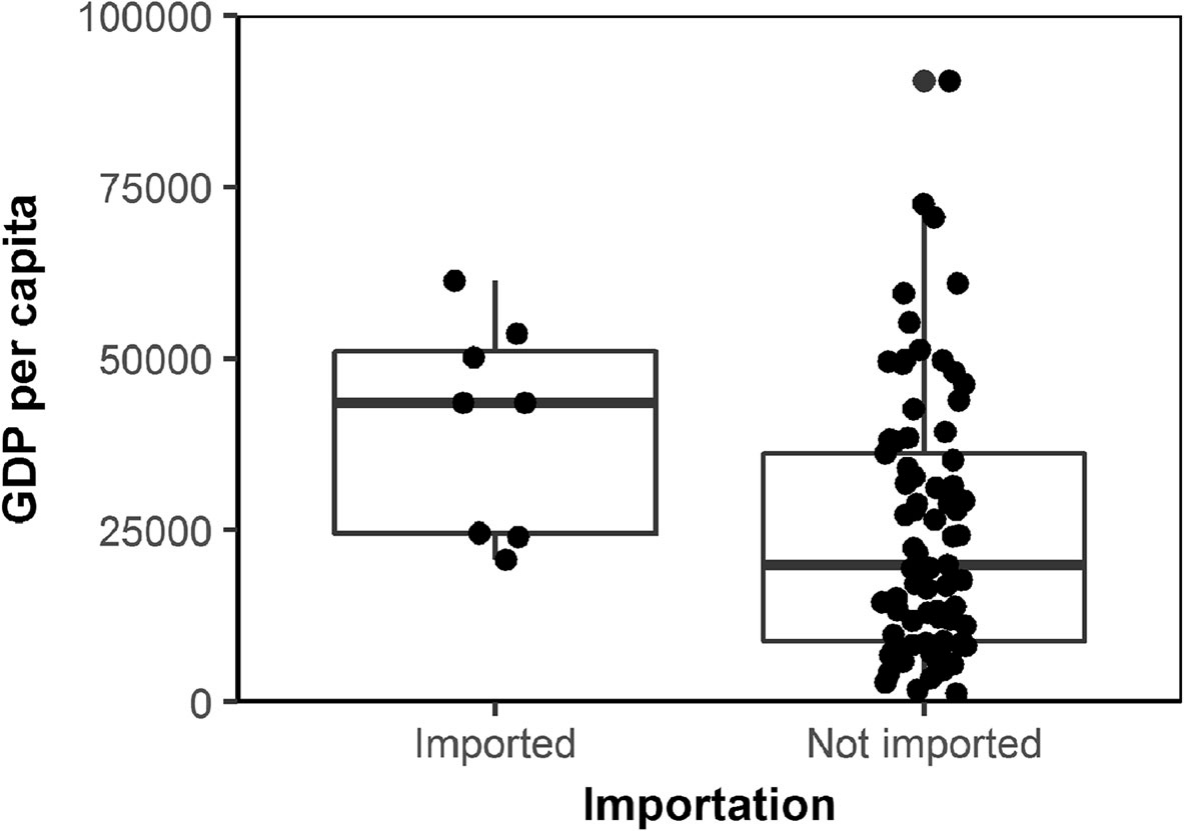
Comparison of gross domestic product (GDP) per capita by importation of yellow fever during 2017–18 epidemic (*n* = 86). GDP per capita is compared between countries with and without imported cases (*n* = 8 and 78, respectively) that were included in our analysis. Mid bold line in the hinges represents median value. The lower and upper hinges correspond to the first and third quartiles. The upper whisker extends from the hinge to the largest value no further than 1.5 times interquartile range, and the lower whisker extends from the hinge to the smallest value at most 1.5 times interquartile range

Figure 1 shows the epidemic curve of 2017–18 epidemic in Brazil as a function of the week of report [9]. The highest incidence was reported in Week 3 of 2018 followed by mostly a monotonic decline in incidence, and the incidence has greatly waned by Week 17, 2018. Figure 2 shows the comparison of GDP per capita by countries with and without imported cases. GDP per capita of countries with imported cases (*n* = 8) was 40,213 US dollars (95% CI: 25,614, 54,811), while that of countries without imported cases and with direct flight link from Brazil (*n* = 78) was 26,368 US dollars (95% CI: 21,593, 31,043). It appears that countries with imported cases have significantly greater GDP per capita than those without imported cases (*t* = 2.34, *p* = 0.04 by Student t-test).

**Fig. 3:**
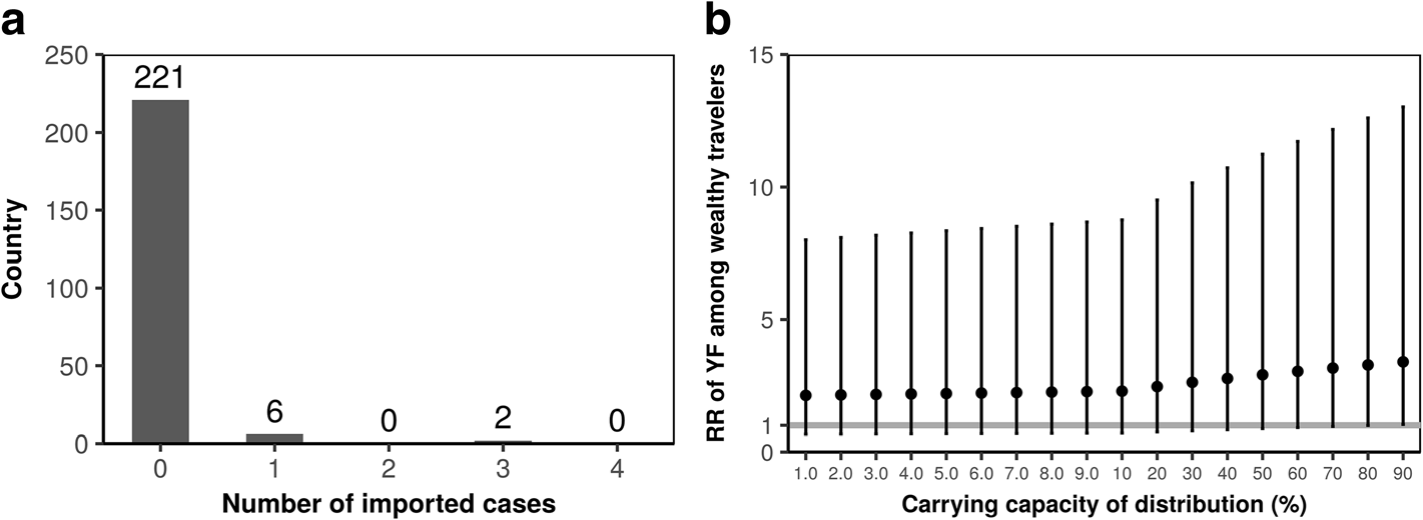
The risk of yellow fever among travelers visiting Brazil. **a** Observed distribution of the number of imported cases of yellow fever from Brazil. As of 8 May 2018, a total of 12 cases were diagnosed in 8 countries. No country experienced 4 or more imported cases. **b** Sensitivity of the relative risk of yellow fever among travelers to the assumed maximum vaccination coverage (horizontal axis). Vertical axis stands for the relative risk of importation among countries above median GDP per capita compared with remaining countries. Filled circles represent the maximum likelihood estimates and whiskers extend to upper and lower 95% confidence intervals as computed from the profile likelihood. Horizontal grey line indicates the value of 1.0

In total, 12 imported cases were reported from 8 different countries (Fig. 3a). Other 78 countries with inbound data to Brazil were included in the following analysis. Known vaccinated fractions were as follows: Trinidad and Tobago (0.96), Panama (0.73), Argentina (0.94), Colombia (0.91), Suriname (0.89), Peru (0.90), Venezuela (0.87), Ecuador (0.78), Paraguay (0.80), Guyana (0.95), Bolivia (0.89), Angola (0.80), Nigeria (0.74), Ghana (0.89) and Kenya (0.78). Assuming *k* = 0.10, travelers from wealthier fraction of countries were *a* = 2.3 (95% CI: 0.7, 8.6) times more likely to be infected with yellow fever compared with countries below median GDP per capita. Compared with an assumption that the risk of infection was determined by the relative population size of epidemic locations (i.e., 3 states with substantial number of cases) to the entire Brazil (i.e. eq. (1) with *q* = 1.0), the assumption of which was employed elsewhere [16], even countries below median GDP per capita experienced *q* = 2.5 (95% CI: 0.8, 5.9) times greater risk of yellow fever.

Figure 3b shows the result from sensitivity analysis. Varying an uncertain parameter *k*, i.e., the possible maximum value of the vaccination coverage among travelers from countries without routine immunization against yellow fever, the estimates of *q*_i_ did not vary greatly. The relative risk *a* of yellow fever among wealthier countries compared with countries with second half of GDP per capita ranged from 2.1 with *k* = 0.01 to 3.4 with *k* = 0.90. Also, compared with random assignment of travelers’ destination by relative population size of States, countries below median GDP per capita experienced *q* = 2.5 (*k* = 0.01) to 2.8 (*k* = 0.90) times greater risk of yellow fever.

## Discussion

Unlike the epidemic from 2016 to 17 in Brazil that was mostly restricted to the States of Minas Gerais and Espirito Santo, the epidemic from 2017 to 18 mainly involved São Paulo and Rio de Janeiro and resulted in multiple international disseminations of imported cases. To understand possible mechanisms behind this observation and also to consider possible countermeasures, the present study explored the distribution of imported cases from Brazil. Employing a statistical model, we described the risk of observing imported case, jointly estimating the relative risk of travelers by the extent of wealth (or GDP per capita) and the relative difference compared with random assignment of travelers’ destination within Brazil. As a result, it appears that wealthier travelers were at 2.1 to 3.4 times greater risk of infection than others. Moreover, even among countries with lower half of GDP per capita, the risk was 2.5 to 2.8 times greater than that with the assumption that the relative risk within Brazil is determined by regional population size.

There are two take home messages. First, we have shown that countries with wealthier GDP per capita appeared to be more often infected. The finding is in line with the fact that the imported cases arose from a holiday spot in Ilha Grande, municipality of Angra do Reis, State of Rio de Janeiro [9, 10]. It also indicates that travelers’ local destination and behavior at high risk of infection are likely to act as a key determinant of the heterogeneous risk of importing case. It is advised to well inform travelers over the ongoing geographic foci of transmission, and if it appears unavoidable to visit tourist destination that has the history of producing imported cases, travelers must be strongly advised to receive vaccination in advance.

Second, we found that even non-wealthy countries were at 2.5–2.8 times greater risk of importing yellow fever case as compared with a common modeling assumption (i.e., *q* = 1.0 in eq. (1)) that the destination-specific risk of infection is proportional to the relative population size of the destination to the entire country. In the case of Brazil, undoubtedly the major tourist destinations of international travelers are São Paulo and Rio de Janeiro. To precisely estimate the risk of infection among travelers, it is ideal to track down travel patterns within Brazil more in detail. A big challenge to achieve precise estimation of the risk in the future would be to quantify such risk in a finer spatial scale using limited mobility information among travelers.

Four limitations must be noted. First, the notification of yellow fever cases is undoubtedly biased by the extent of ascertainment. Thus, even though we found that travelers from countries with greater GDP per capita were at greater risk of yellow fever, the finding could partly reflect better ascertainment of cases in wealthier countries compared with the reminder. Second, we were not able to account for spatial risk of infection in a finer scale. As of 8 May 2018, the transmission has not been established within the city of Rio de Janeiro [20], and thus, such risk at greater precision must be communicated with risk map, as it was published elsewhere [21–23]. Third, the vaccination coverage among travelers from countries without routine yellow fever immunization was assumed to be proportional to GDP per capita. In the present study, our estimates were not sensitive to the ceiling of the vaccination coverage, *k*, but this strong assumption needs to be validated through empirical observation in the future. Fourth, the lower confidence bound of our relative risk estimates were smaller than the value of 1 (e.g., with *k* = 0.10, the lower 95% CI of *a* was 0.6), and the sample size was not substantial. This is due to limited number of countries that imported yellow fever. With greater sample size in a future follow-up study, uncertainties would be reduced, and moreover, our conclusions would be strengthened.

Apart from these future tasks for finer estimation of the risk of infection among travelers, we believe that our study successfully quantified the relative risk of infection by GDP per capita and also compared with the risk that rests on population-size specific assumption of travelers’ destination. Micro-geographic information of imported cases should be effectively shared with travelers for communication and prevention purposes.

## Conclusions

Travelers from wealthier countries were at elevated risk of yellow fever, allowing us to speculate that travelers’ local destination and behavior at high risk of infection are likely to act as a key determinant of the heterogeneous risk of importation. As part of important messages as derived in real time studies [19, 24–27], it is advised to inform travelers over the ongoing geographic foci of transmission, and if it appears unavoidable to visit tourist destination that has the history of producing imported cases, travelers must be strongly advised to receive vaccination in advance.

## Abbreviations

CI: Confidence interval
GDP: Gross Domestic Product
WHO: World Health Organization
